# CMOS-Compatible Fabrication Module for Sub-100 nm TiN and TaN Pillar Electrodes for Carbon Nanotube Test Structures

**DOI:** 10.3390/nano16060357

**Published:** 2026-03-14

**Authors:** Guohai Chen, Takeshi Fujii, Takeo Yamada, Kenji Hata

**Affiliations:** Nanocarbon Material Research Institute, National Institute of Advanced Industrial Science and Technology (AIST), Tsukuba Central 5, 1-1-1 Higashi, Tsukuba 305-8565, Ibaraki, Japan

**Keywords:** pillar electrode, carbon nanotube, CNT, CRAM, memory

## Abstract

We report a versatile, CMOS-compatible fabrication module for sub-100 nm TiN and TaN pillar electrodes, a key building block for sandwich-type test structures. As a demonstration, the electrodes were integrated into carbon nanotube-based nonvolatile random-access memory (CRAM) test structures. High-resolution hydrogen silsesquioxane (HSQ) masks defined by electron beam lithography were transferred into TiN films using optimized Ar/Cl_2_ inductively coupled plasma reactive ion etching. Optical emission spectroscopy was used for real-time endpoint detection, ensuring precise etch control. The process achieved a TiN-to-HSQ selectivity of ~1.6 and reproducible nanoscale features with smooth sidewalls and an average taper angle of ~77°. Buffered hydrogen fluoride treatment effectively removed residual HSQ, revealing sharp TiN features and preserving pillar geometry. Atomic force microscopy (AFM) confirmed pillar height and profile fidelity, while conductive AFM verified electrical conductivity after planarization. The module was further demonstrated through the fabrication of TiN pillar arrays, TaN pillars, and sub-100 nm TiN line arrays. A CRAM test structure incorporating TiN pillars exhibited preliminary switching, indicating that both the test structure and fabrication process are feasible. This fabrication module provides a reproducible platform for nanoscale TiN and TaN electrodes, supporting laboratory-scale research and providing a pathway toward future integration of emerging memory and nanoelectronic technologies.

## 1. Introduction

The rapid advancement of memory technologies, together with the rapid growth of artificial intelligence (AI) and machine learning (ML), has intensified the demand for high-performance, low-latency, and energy-efficient memories capable of handling massive data processing workloads. Meeting these requirements for next-generation nonvolatile memories increasingly relies on precisely engineered nanoscale electrode architectures [1,2]. In particular, pillar and line electrodes form the core of a wide range of sandwich-type memory devices, including resistive random-access memory (ReRAM) [3,4], phase-change memory (PCM) [5], ferroelectric RAM (FeRAM) [6], and magnetic RAM (MRAM) [7]. In such devices, electrode geometry and reproducibility are crucial in controlling current confinement, switching uniformity, and scalability. Sub-100 nm pillar electrodes are therefore valuable not only for industrial integration but also for laboratory research, where they provide reproducible testbeds to evaluate new switching materials, probe electrode-interface interactions, and explore scaling effects without requiring full device fabrication. By serving as a standardized platform, these nanoscale electrode structures bridge exploratory research and industrial process flows, making them critical for both academic laboratory-scale studies and pre-industrial prototyping of emerging memory technologies.

Within this broader context, carbon nanotube (CNT)-based nonvolatile random-access memory (CRAM/NRAM) has emerged as one of the promising candidates for next-generation nonvolatile memory devices owing to its fast-switching speed, low power consumption, and high endurance [8,9,10,11,12,13,14,15,16]. In these memory devices, switching is reported to originate from rearrangements at CNT junctions, which enable stable high- and low-resistance states [9,17]. Recent progress in data-driven materials research has further shown that AI/ML can accelerate CNT device optimization and process development, potentially reducing experimental cycles and uncovering new structure–property relationships [18,19,20,21,22]. Nevertheless, the full fabrication of such memory devices in semiconductor fabs remains highly complex. In the case of CRAM devices, fabrication requires long, multi-step process flows, such as dielectric deposition, CNT slurry integration, multiple thermal annealing, planarization, passivation, and multi-layer electrode patterning [10,13]. The extended fabrication cycle delays experimental feedback, resulting in slow process optimization and substantial resource consumption.

To address this bottleneck, we employ a streamlined fabrication approach based on simplified test structures that capture the essential process modules while significantly reducing overall fabrication time. This strategy enables rapid feedback on key fabrication steps, lowers material usage and processing overhead, and accelerates the screening of CNT slurries, electrode designs, and process parameters toward full CRAM device realization.

A central challenge in developing such streamlined test structures, as well as in memory research more broadly, is the realization of nanoscale electrodes that can interface effectively with active switching layers, such as CNT networks in CRAM devices, while maintaining compatibility with CMOS process flows. Titanium nitride (TiN) and tantalum nitride (TaN) are particularly attractive candidates due to their chemical stability, mechanical robustness, good conductivity, and widespread use in semiconductor manufacturing [23,24,25]. Both materials have been widely used as gate electrodes and diffusion barriers in CMOS [23,25,26,27,28], highlighting their industrial relevance and suitability as practical laboratory-scale electrode platforms.

Nevertheless, fabricating reliable sub-100 nm TiN and TaN pillar/line features with high fidelity and reproducibility remains technically demanding. A major challenge lies in the dry etching of TiN and TaN. Numerous chemistries have been investigated, including HBr/Cl_2_ [25], Cl_2_/H_2_ [27], BCl_3_/Cl_2_/Ar [28], CF_4_/O_2_ [29], CF_4_/BCl_3_/N_2_ [30], Cl_2_/Ar [31,32], Cl_2_/N_2_ [33], and HBr/O_2_ [34]. These works reveal key trade-offs between etch rate, anisotropy, and selectivity to SiO_2_. For example, TiN etching is typically ion-flux limited and often requires elevated bias power to achieve anisotropic profiles [33,35]. The addition of Ar enhances physical sputtering but can lower selectivity, while oxygen or boron additives alter surface passivation layers, influencing taper angle and residue formation [28,34,35]. Recent work demonstrates that plasma atomic layer etching can achieve near-atomic-scale control of TiN at low temperatures [36], though throughput and integration challenges remain. Refinements in selective etching have further improved outcomes. For TiN, the incorporation of N_2_ into Cl_2_/Ar plasmas significantly reduces nonvolatile sidewall residues while achieving high selectivity (~50) over SiO_2_ [37]. For TaN, O_2_/BCl_3_/Cl_2_/Ar ICP chemistries achieve etch rates up to 172.7 nm/min and selectivity of ~6.3 over SiO_2_, though sidewall passivation and Ta-O_x_/Ta-Cl_x_ by-products remain concerns [38]. These advances highlight the importance of precisely tuned plasma chemistries for fabricating reliable sub-100 nm nitride structures.

In addition, reliable electrical contact of sub-100 nm vertical pillars presents another critical challenge. Previous work has demonstrated hydrogen silsesquioxane (HSQ)-based planarization and etch-back or atomic force microscopy (AFM) nano-indentation strategies to contact vertical Si nano-pillars [39]. However, the integration of such contacting approaches with CMOS-compatible metal-nitride electrodes and functional switching layers has been less explored.

In this work, we report a CMOS-compatible fabrication module for sub-100 nm TiN and TaN pillar electrodes integrated into CRAM test structures. HSQ resist was patterned by electron beam lithography (EBL) and transferred into TiN or TaN films using an optimized inductively coupled plasma reactive ion etching (ICP-RIE) process based on Ar/Cl_2_ chemistry. Optical emission spectroscopy (OES) was used to monitor the 506 nm emission line for in situ endpoint detection, ensuring precise etch control. The process produced pillars with well-defined, smooth sidewalls, and an average taper angle of ~77°. AFM confirmed pillar heights across fabrication stages, while conductive AFM further verified localized electrical conduction through TiN pillars embedded in the SiO_2_ dielectric after planarization. Importantly, the CRAM test structures fabricated with these electrodes displayed preliminary switching behavior, demonstrating the structural and functional feasibility of the developed process module. By establishing a streamlined fabrication method capable of delivering rapid feedback on CRAM test structures, this work provides a robust and versatile platform for various sandwich-type architectures such as ReRAM, PCM, FeRAM, and MRAM, offering a laboratory-scale environment for accelerating the discovery, optimization, and integration of next-generation memory technologies.

## 2. Experimental

### 2.1. Fabrication Process

The Si/SiO_2_ substrate was sequentially cleaned in acetone and isopropanol (from Fujifilm Wako Pure Chemical, Osaka, Japan) and then deionized (DI) water prior to film deposition. All reagents were used as received. The following detailed process conditions were applied.

TaN film deposition: A Ta target (99.99%, from CHEMISTON, Kawajima, Saitama, Japan) was sputtered in a gas mixture of 9 sccm Ar and 1 sccm N_2_ at a pressure of 0.5 Pa using an RF power of 200 W (Shibaura CFS-4EP-LL, Yokohama, Kanagawa, Japan). Deposition was carried out for 50 min, targeting a thickness of 500 nm. The measured film thickness was 498.0 ± 2.9 nm.

TiN film deposition: A Ti target (99.99%, from CHEMISTON, Kawajima, Saitama, Japan) was sputtered using 5 sccm Ar and 5 sccm N_2_ at 0.5 Pa with an RF power of 200 W (Shibaura CFS-4EP-LL, Yokohama, Kanagawa, Japan). The deposition time was 64 min, targeting a thickness of 150 nm. The measured film thickness was 153.6 ± 4.5 nm.

HSQ spin-coating: The substrate was first treated with hexamethyldisilazane (HMDS, from Tokyo Ohka Kogyo, Kawasaki, Kanagawa, Japan, 30 s at 3200 rpm) as an optional adhesion promotion step, followed by HSQ coating (XR-1541-006 resist from DuPont, Midland, MI, USA, 60 s at 3200 rpm) using a spin coater (Mikasa MS-B150, Tokyo, Japan). A soft bake was performed at 180 °C for 3 min.

EBL: Patterning was performed with an accelerating voltage of 130 kV, beam current of 150 pA, and a dose of 2475 µC/cm^2^ (Elionix ELS-F130AN, Hachioji, Tokyo, Japan). Development was carried out in 2.38% tetramethylammonium hydroxide (TMAH, from Tokuyama Corporation, Shunan, Yamaguchi, Japan) for 90 s, followed by DI water rinse and post-bake at 180 °C for 5 min.

ICP-RIE etching: TiN and TaN were etched using a gas mixture of 100 sccm Ar and 20 sccm Cl_2_ at a pressure of 1 Pa, with an ICP power of 500 W, a bias power of 80 W, and a stage temperature of 20 °C (Samco RIE-400iPS, Shinagawa, Tokyo, Japan).

BHF treatment: Residual HSQ was removed by soaking the sample in ultra-high purity buffered hydrogen fluoride solution (BHF, LAL200 from Stella Chemifa, Osaka, Japan) for 2 min, followed by DI water rinse.

SiO_2_ dielectric deposition: Plasma-enhanced chemical vapor deposition was performed using 5 sccm TEOS and 95 sccm O_2_ at a pressure of 40 Pa, with a RF power of 250 W, a substrate temperature of 350 °C, and a deposition of 6 min (Samco PD-20SS, Shinagawa, Tokyo, Japan).

### 2.2. Characterizations

In situ optical emission spectroscopy (OES, Ocean Insight HR4Pro, Tokyo, Japan) [33,34,35] was used during the Cl_2_-based ICP-RIE etching process to monitor the etching endpoint. Scanning electron microscopy (SEM, Hitachi FE-SEM SU8200, Tokyo, Japan) was conducted to characterize surface morphology and structural profiles of the pillars. AFM (Bruker Dimension Fastscan, Yokohama, Kanagawa, Japan) provided precise height measurements and 2D/3D image profiling of fabricated structures. Conductive AFM [40,41] was further applied to verify the electrical conduction of TiN pillars after planarization process.

### 2.3. Electrical Measurements

The switching performance of the fabricated CRAM test structure was assessed using a semiconductor parameter analyzer (Keysight B1500A, Hachioji, Tokyo, Japan) and a write voltage sweep from 0 V to +3 V, then from +3 V back to −3 V and finally from −3 V back to 0 V with a step of 0.1 V. The read current was measured by applying a small read voltage of 0.5 V. A compliance current of 50 µA was imposed during the set operation to prevent test structure breakdown.

## 3. Results and Discussion

**Figure 1:**
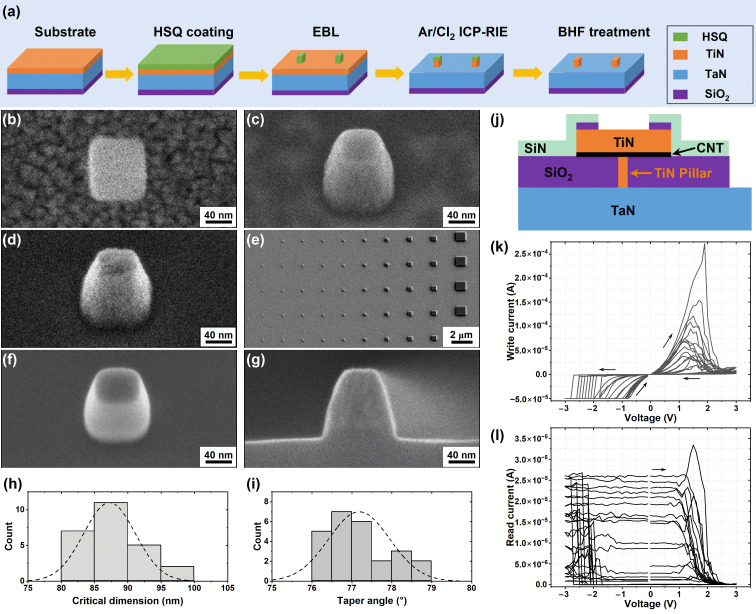
(**a**) Schematic illustration of the pillar fabrication process. SEM images showing the pillars at different fabrication stages: (**b**) HSQ pillar patterned by EBL, (**c**) TiN pillar after Cl_2_-based ICP-RIE etching, and (**d**) TiN pillar after removing HSQ mask by BHF treatment. Tilt angle: 31°. (**e**) SEM image of a TiN pillar array with lateral widths of 40, 60, 80, 90, 100, 200, 300, 400, 500, and 1000 nm (left to right). (**f**) SEM image of a TaN pillar fabricated using the same process. Tilt angle: 31°. (**g**) Cross-sectional SEM image of a TiN line fabricated by the same process. SEM observations were carried out at an accelerating voltage of 3 kV with a working distance of approximately 8.3 mm. Histograms showing (**h**) the critical dimension of nominal 90 nm TiN pillars and (**i**) the taper angle. (**j**) Schematic of the CRAM test structure fabricated using TiN pillar electrode and (**k**,**l**) its representative electrical performance. Arrows denote the voltage sweep direction.

We begin by introducing the process flow of pillar fabrication as the core enabling step of our CRAM test structures. In contrast to full CRAM device fabrication, which can require weeks to months due to complex processes, these test structures can be completed within a few days, providing rapid feedback. Semiconductor fab-friendly materials, TiN and TaN, were chosen for their backend compatibility and excellent electrical/thermal stability. As depicted in Figure 1a, the process starts with a substrate coated with a sputtered TaN layer acting as the common bottom electrode layer (BEL), followed by a sputtered TiN film for the pillar layer. HSQ, a high-resolution negative-tone resist [42], is spin-coated and then patterned by EBL to define nanoscale pillar masks. The TiN film is then etched through an Ar/Cl_2_-based ICP-RIE process. Finally, the remaining HSQ mask layer is removed through a wet etch in BHF, which completes the TiN pillar fabrication module.

Next, we evaluated the morphologies of pillars during three most critical steps. First, after EBL, the resulting HSQ pillars were examined by SEM (Figure 1b). After development, the unexposed area was completely removed, revealing the underlying TiN surface, while the exposed area remained as well-defined square HSQ pillars, that serve as masks for the next etching step. The TiN layer was then etched through a finely tuned Ar/Cl_2_-based ICP-RIE process, producing moderately tapered TiN pillars with clearly defined smooth sidewalls (Figure 1c). To finalize the structure, residual HSQ was removed by soaking in a BHF solution, which effectively cleaned the surface while revealed the crystalline TiN features (Figure 1d).

These optimized process conditions enable the formation of a wide range of nanoscale fine features. First, Figure 1e shows a TiN pillar array with feature sizes spanning from 1000 nm down to 40 nm, although the smallest dimension still requires additional optimization for better resolution. The smallest pillars appear less well defined, likely due to tool resolution limits, electron beam proximity effects and aspect ratio–dependent etching [43]. Optimizing exposure dose and ICP bias may further increase resolution while maintaining sidewall smoothness. In addition, the same recipe was extended to TaN pillars, yielding well-defined pillar structures with smooth sidewalls, as shown in Figure 1f. TaN etching typically suffers from surface oxide formation and halogen–oxygen complex residues, which can degrade anisotropy and commonly require pre-removal or O_2_/BCl_3_ additives. Our Ar/Cl_2_ chemistry, free of BCl_3_ or O_2_, avoids such complex formation, while the high Ar fraction provides sufficient physical sputtering to continuously remove native oxides during etching. This enables clean, vertical sidewalls without separate oxide-clean steps, in line with prior findings that Cl_2_/Ar ICP can etch TaN anisotropically given adequate ion energy, whereas chemistries lacking strong sputtering often require multi-step cleans [31,38]. Furthermore, beyond isolated pillars, the method also allows fabrication of line arrays. Figure 1g presents a cross-sectional SEM image of a 100 nm-wide TiN line produced using the same etching conditions, demonstrating the versatility of the developed process module. Dimensional control was further evaluated for nominal 90 nm TiN pillars (Figure 1h). The measured critical dimension was 87.2 ± 3.9 nm (n = 25). The taper angle, calculated from the measured top width, bottom width, and pillar height, averaged 77.2 ± 0.8° (n = 25) (Figure 1i). Considering that the fabrication was performed using shared and legacy facilities, these results demonstrate acceptable dimensional control and profile fidelity. Although the sidewalls are not perfectly vertical, they are smooth and free of pronounced footing or microtrenching, indicating a well-balanced ion-assisted Cl_2_-based etching regime. For the intended test-structure platform, this level of verticality provides adequate dimensional stability and electrical reliability, while further optimization remains feasible if more aggressive scaling is required.

Using these TiN pillars as an important feature element, a CRAM test structure was fabricated (Figure 1j). The main fabrication steps include SiO_2_ dielectric deposition, planarization (chemical mechanical polishing, CMP), CNT slurry spin-coating, thermal annealing, photolithography and etching to define test structure areas (pucks), SiN passivation, and formation of metal contacts to the top electrode (TE) and bottom electrode (BE) through photolithography, etching, and lift-off processes. The CNT film is a critical component of the test structure. Single-walled CNTs (a mixture of semiconducting and metallic CNTs) with typical diameter of 0.9–2 nm and lengths ranging from several tens of nanometer to several hundred micrometers were used. These CNT were converted into a semiconductor-grade aqueous formulation through a series of purification processes, including acid functionalization, metal removal, amorphous carbon removal, defect removal and quality control to ensure compatibility with wet processing and coating performance. The resulting slurry was spin-coated onto the wafer. The final CNT film forms a ~30 nm thick random CNT network [10]. After being thermally annealed in nitrogen, an electrical measurement confirmed the preliminary switching functionality of this CRAM test structure (Figure 1k,l). Clear bipolar resistive switching behavior was observed. During the write-voltage sweep, the test structure reset from the low-resistance state (LRS) to the high-resistance state (HRS) in the positive voltage range of +1.2 to +1.8 V. When the sweep continued toward negative bias, it set back to the LRS in the range of −1.7 to −2.7 V. The corresponding read-current mapping confirmed transitions between distinct resistance states throughout the sweep. The ON/OFF ratio exceeded 2000 in the early cycles and decreased to ~100 after approximately 26 cycles. Although variations are observed and these measurements represent preliminary feasibility results, the overall results are encouraging for this CRAM test structure. In this work, we focus primarily on the fabrication of the pillar electrodes. Detailed information including fabrication process of this test structure and its performance, such as reproducibility, endurance, retention, measurement protocol, and switching and failure mechanisms are ongoing and will be presented in a subsequent paper. Accordingly, the present results are intended to demonstrate structural and process feasibility rather than fully optimized device performance.

Together, these results demonstrate the robustness and versatility of the optimized ICP-RIE recipe, enabling reproducible fabrication of TiN and TaN nanostructures across a wide size range without the need for complex multi-gas etching chemistries.

**Figure 2:**
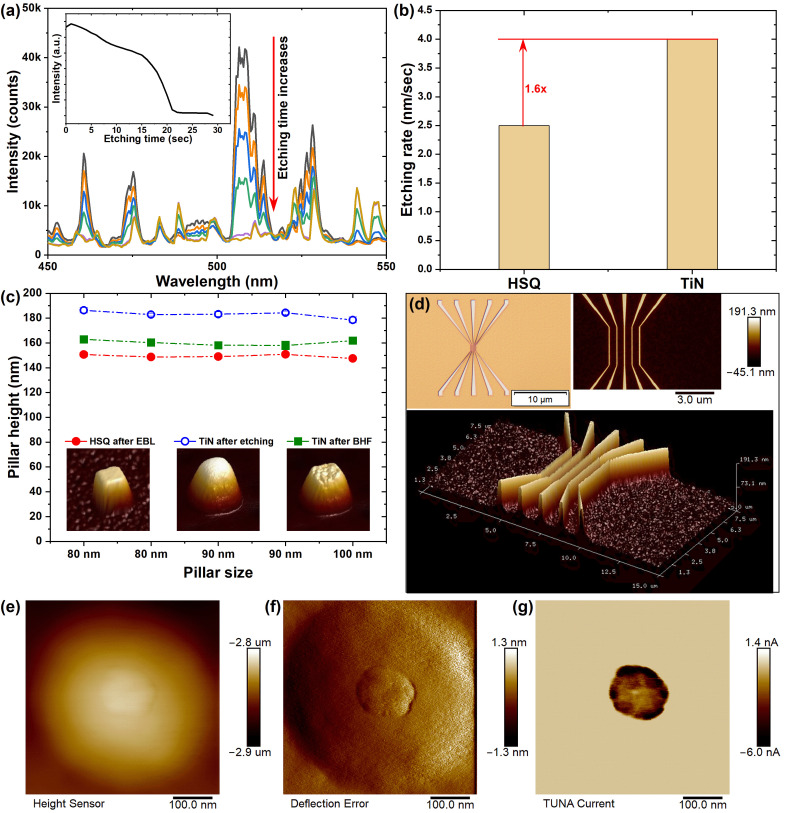
(**a**) Optical emission spectra recorded during ICP-RIE along with etching time, used for endpoint determination. Inset: Representative OES temporal evolution of the 506 nm emission intensity during etching of a thinner TiN film. (**b**) Etching rates of HSQ and TiN. (**c**) AFM-measured pillar heights at different process stages. Insets: corresponding 3D AFM profiles. (**d**) AFM measurement of a five-line TiN array: line width of the central part is 90 nm. Conductive AFM characterization of a TiN pillar after planarization process: (**e**) topography image, (**f**) deflection error image, and (**g**) current image confirming electrical conduction.

During the establishment of the etching recipe, OES was used as an in situ diagnostic tool during the ICP-RIE process to determine the TiN film etching endpoint [33,34,35]. Figure 2a presents representative spectral snapshots over a broad wavelength range as a function of etching time. The inset shows the temporal evolution of the 506 nm emission line intensity during etching. As illustrated, the intensity initially remained high and gradually decreased as etching progressed, eventually approaching the background level, which indicates the completion of the TiN removal. Continuous monitoring of this emission line enabled accurate real-time endpoint detection, minimizing both over-etching and under-etching.

The TiN-to-HSQ etching selectivity was next evaluated (Figure 2b). Cross-sectional SEM analyses for varying etching durations yielded etch rates of approximately 4 nm/s for TiN and 2.5 nm/s for HSQ, corresponding to a selectivity of ~1.6. Although not exceptionally high, this selectivity is sufficient for maintaining pattern fidelity and process margin in sub-100 nm fabrication. These values align closely with prior reports for TiN dry etching in similar Cl_2_-based ICP chemistries. For instance, Tonotani et al. reported a TiN etch rate of 230 nm/min (~3.8 nm/s) with a selectivity to photoresist of 1.73 under Ar/Cl_2_ ICP-RIE conditions [35]. Our results are thus representative of optimized Cl_2_-based anisotropic etching regimes. The measured TiN-to-HSQ selectivity (~1.6) and pillar taper angle (~77°) are characteristic of ion-assisted Cl_2_-based etching in ICP systems under moderate RF bias, where a balance between chemical chlorination and physical ion sputtering ensures effective anisotropic etching without significant microtrenching [28,33]. In this regime, Cl radicals react with Ti to form volatile TiCl_x_ species, while Ar^+^ bombardment enhances both the etch rate and anisotropy by removing surface oxides and suppressing spontaneous isotropic etching. Such behavior is well suited for sub-100 nm structures, as moderate selectivity preserves the mask while avoiding excessive microtrenching or footing. Benchmarking against established data further indicates that our process falls within a robust, industry-compatible etching window suitable for advanced electrode fabrication.

AFM analysis was used to quantify pillar dimensions at different process stages. The measured pillar heights exhibited minimal dependence on pillar width in the range from 80 to 100 nm (Figure 2c). HSQ pillars patterned by EBL showed an average height of 149.6 ± 1.7 nm (n = 25). After Cl_2_ etching, the TiN pillar height, including the remaining HSQ mask layer, increased to 178.0 ± 6.4 nm (n = 15). Subsequent HSQ removal by BHF treatment resulted in a final pillar height of 156.2 ± 3.9 nm (n = 15). The corresponding 3D AFM topography images further confirmed that the pillar geometry was well maintained throughout the fabrication process.

Pattern fidelity for sub-100 nm feature was further evaluated through AFM characterization of a TiN five-line array (Figure 2d). The central line exhibited a well-defined line width of ~90 nm with a uniform spacing of 500 nm, and both 2D and 3D AFM profiles verified that the structures were clearly resolved with no evidence of collapse or distortion. This provides confidence that the etch process can be reliably extended to dense arrays over large die areas without major modification. This is especially important for crossbar-type nonvolatile memory architectures, where cumulative dimensional variations can directly influence switching-voltage distributions, leakage current paths, and array yield.

Finally, conductive AFM measurements were conducted to examine the electrical integrity [40,41] of TiN pillars after the planarization process, a critical step for CRAM test structures. This serves as an early-stage electrical verification tool within our process development flow. By directly probing the fabricated TiN pillars, we can measure current–voltage characteristics at the individual-feature level without completing the full test structure. This capability is critical: it allows functional assessment of electrode conductivity, continuity, and potential leakage paths at the pillar stage, long before integration with the dielectric and top electrode layers. Instead of using outsourced CMP, an in-house dry etch-back process was developed for the planarization [44]. Briefly, the fabricated TiN pillar sample was coated with SiO_2_ dielectric layer and then spin-coated with HSQ, followed by thermal annealing (1 h at 400 °C under 1000 sccm N_2_ flow). A carefully tuned Ar/CF_4_-based ICP-RIE etching recipe was used to etch the HSQ and SiO_2_ dielectric at a similar etching rate, resulting in a planarized surface while selectively exposing the TiN pillar, ready for the next CNT coating [45]. The topography image clearly showed the surface profile of the structure with height variations, revealing the pillar embedded within the SiO_2_ dielectric matrix (Figure 2e). The deflection error image highlighted the fine surface structural features and boundaries (Figure 2f). The current image showed the spatial distribution of current between the conductive AFM tip and the pillar, confirming localized electrical conduction through the TiN pillar. These results validate the feasibility of the etch-back planarization method and confirm the suitability of TiN pillars as electrodes in CRAM test structures.

## 4. Conclusions

In conclusion, we have developed and demonstrated a robust CMOS-compatible fabrication module for nanoscale TiN and TaN pillar structures. The combination of EBL-defined HSQ masks, optimized Ar/Cl_2_ ICP-RIE, and BHF post-treatment enabled the realization of pillars with well-defined profiles, high structural fidelity, and reproducible dimensions. OES-based endpoint detection provided precise control of the etching process, while AFM analyses confirmed pillar heights and shape preservation. Conductive AFM measurements further validated the electrical integrity of TiN pillars after planarization, a critical step for device integration. The versatility of the method was demonstrated by fabricating both isolated pillars and line arrays across a wide dimensional range, including sub-100 nm features. More importantly, although the CRAM test structures presented here exhibited only preliminary switching behavior, they confirm the structural and process feasibility of the developed module. Overall, this CMOS-compatible process module establishes a reliable laboratory-scale platform for rapid process feedback and materials evaluation, while offering a potential pathway toward future industrial integration.

## Data Availability

Data is contained within the article.
